# Supplementary material for: Handy divisions: Hand-specific specialization of prehensile control in bimanual tasks
Source: PLoS One. 2025 Apr 16;20(4):e0321739. doi: 10.1371/journal.pone.0321739 (PMC12002523; doi:10.1371/journal.pone.0321739)
Supplement: S1 Appendix — (DOCX) [file pone.0321739.s001.docx]

**S1 Appendix**

**Cross-recurrence quantification analysis for grip-load coupling**

Although cross-correlational analysis has been effective in capturing gross features of the coupling [1, 2], these methods are not suited to identify subtle temporal variations in the coupling [3, 4, 5]. Therefore, we also used a non-linear method called CRQA to quantify the strength of the grip-load coupling.

CRQA quantifies how two processes unfold and interact over time by computing how often their time series come close to each other in a common reconstructed phase space [6]. This technique proceeds in three steps. First, a common phase space for both time series is constructed using time-delayed versions of both series [7]. Second, instances when the phase-space trajectories of the two time series are within a pre-defined distance, known as radius, from each other are identified. These instances, called cross recurrence points, are used to create a cross recurrence plot. Third, the patterns in the cross recurrence plots are characterized by computing the appropriate outcome measures.

To construct the phase space and the cross recurrence plot for the digit forces, the maximum (L_∞_) norm [7] was used. The three input parameters required for this technique were identified using established recommendations: the embedding dimension [8], time delay [9], and radius [10]. These parameters were computed using all trials across all participants. The embedding dimension was set to 5, and the time delay was set to 23 samples (115 ms). The augmented Dickey-Fuller test [11, 12] identified non-stationarity in the grip force time series. Therefore, the radius was selected adaptively for each trial to ensure a fixed recurrence rate of 5% [4, 13].

We consistently observed vertical line structures in the cross-recurrence plots (Fig 6 in main text). This suggest that the state of one process does not change or changes slowly relative to the state of the other process, i.e., one time series gets trapped at a location while the other deviates from that location [14]. This implies the coupling between the two processes is intermittent [3, 15], and the degree of intermittency can be used to quantify the strength of the coupling. In particular, the strength of the coupling is inversely proportional to the intermittency, which, in turn, can be quantified using characteristic length of the vertical lines. This characteristic length was computed using trapping time, which is the average length of all the vertical lines in a plot [15, 16].

**Referernces**

1. Flanagan JR, Wing AM. Modulation of grip force with load force during point-to-point arm movements. Exp Brain Res. 1993;95(1):131-43. Epub 1993/01/01. PubMed PMID: 8405245.

2. Gao F, Latash M, Zatsiorsky V. Internal forces during object manipulation. Experimental Brain Research. 2005;165(1):69-83. doi: 10.1007/s00221-005-2282-1.

3. Grover FM, Lamb M, Bonnette S, Silva PL, Lorenz T, Riley MA. Intermittent coupling between grip force and load force during oscillations of a hand-held object. Experimental Brain Research. 2018;236(10):2531-44. doi: 10.1007/s00221-018-5315-2.

5. Grover FM, Nalepka P, Silva PL, Lorenz T, Riley MA. Variable and intermittent grip force control in response to differing load force dynamics. Experimental Brain Research. 2019;237(3):687-703. doi: 10.1007/s00221-018-5451-8.

5. Naik A, Ambike S. The coordination between digit forces is altered by anticipated changes in prehensile movement patterns. Exp Brain Res. 2020;238(5):1145-56. Epub 20200330. doi: 10.1007/s00221-020-05783-1. PubMed PMID: 32232541.

6. Webber CL, Jr., Zbilut JP. Dynamical assessment of physiological systems and states using recurrence plot strategies. J Appl Physiol (1985). 1994;76(2):965-73. Epub 1994/02/01. doi: 10.1152/jappl.1994.76.2.965. PubMed PMID: 8175612.

7. Marwan N, Romano C, M., Thiel M, Kurths J. Recurrence plots for the analysis of complex systems. Physics Reports. 2007;438(5-6):237-329. doi: https://doi.org/10.1016/j.physrep.2006.11.001.

8. Abarbanel H, Brown R, Sidorowich J, Tsimring L. The analysis of observed chaotic data in physical systems. Rev Mod Phys. 1993;65:1331-92. doi: 10.1103/RevModPhys.65.1331

9. Hasson CJ, Van Emmerik RE, Caldwell GE, Haddad JM, Gagnon JL, Hamill J. Influence of embedding parameters and noise in center of pressure recurrence quantification analysis. Gait Posture. 2008;27(3):416-22. Epub 2007/07/03. doi: 10.1016/j.gaitpost.2007.05.010. PubMed PMID: 17604174.

10. Zbilut JP, Thomasson N, Webber CL. Recurrence quantification analysis as a tool for nonlinear exploration of nonstationary cardiac signals. Med Eng Phys. 2002;24(1):53-60. Epub 2002/03/14. PubMed PMID: 11891140.

11. Dickey DA, Fuller WA. Distribution of the estimators for autoregressive time series with a unit root. Journal of the American statistical association. 1979;74(366a):427-31.

12. Dickey DA, Fuller WA. Likelihood ratio statistics for autoregressive time series with a unit root. Econometrica: journal of the Econometric Society. 1981:1057-72.

13. Webber JCL, Marwan N. Recurrence Quantification Analysis Theory and Best Practices: Cham : Springer International Publishing : Imprint: Springer; 2015.

14. Marwan N, Webber CL. Mathematical and Computational Foundations of Recurrence Quantifications. In: Webber CL, Marwan N, editors. Recurrence Quantification Analysis Theory and Best Practices. Switzerland: Springer International Publishing; 2015.

15. Marwan N, Wessel N, Meyerfeldt U, Schirdewan A, Kurths J. Recurrence-plot-based measures of complexity and their application to heart-rate-variability data. Phys Rev E Stat Nonlin Soft Matter Phys. 2002;66(2 Pt 2):026702. Epub 2002/09/21. doi: 10.1103/PhysRevE.66.026702. PubMed PMID: 12241313.

16. Grover FM, Schwab SM, Silva PL, Lorenz T, Riley MA. Flexible organization of grip force control during movement frequency scaling. J Neurophysiol. 2019;122(6):2304-15. Epub 2019/10/17. doi: 10.1152/jn.00416.2019. PubMed PMID: 31618100.
