# Supplementary material for: Handy divisions: Hand-specific specialization of prehensile control in bimanual tasks
Source: PLoS One. 2025 Apr 16;20(4):e0321739. doi: 10.1371/journal.pone.0321739 (PMC12002523; doi:10.1371/journal.pone.0321739)
Supplement: S2 Appendix — (DOCX) [file pone.0321739.s002.docx]

**S2 Appendix**

**Complexity of the excursions of the stabilized object**

The stabilized object displayed some excursions. We assessed whether these excursions were more complex in one of the tasks. We computed sample entropy of the displacement of the stabilized object in the mediolateral and the vertical directions as the measure of complexity. The time series was divided into vectors of length *m* (set to m=3), and each vector was compared with subsequent vectors for repeating elements within a specified tolerance *r* (set to *r* = 2) [1]. Such subsequent comparisons are repeated with vectors of length *m*+1. Sample entropy was then computed as the negative natural logarithm of the conditional probabilities of these two comparisons. A higher sample entropy implies higher complexity.

We pooled sample entropy values across hands for the regular and the irregular tasks. Two-sample t tests revealed that the sample entropy was higher (t_(23)_ = 11.1; *p* < 0.01) during irregular (0.13±0.01) than the regular motion (0.09±0.01) in the mediolateral direction. Likewise, the sample entropy was higher (t_(23)_ = 12.5; *p* < 0.01) during irregular (0.12±0.01) than irregular motion (0.08±0.01) in the vertical direction.

Therefore, the excursions of the stabilized object were more complex during the irregular task.

**Referernces**

1. Ramdani S, Seigle B, Lagarde J, Bouchara F, Bernard PL. On the use of sample entropy to analyze human postural sway data. Med Eng Phys. 2009;31(8):1023-31. Epub 2009/07/18. doi: 10.1016/j.medengphy.2009.06.004. PubMed PMID: 19608447.
