# Supplementary material for: Handy divisions: Hand-specific specialization of prehensile control in bimanual tasks
Source: PLoS One. 2025 Apr 16;20(4):e0321739. doi: 10.1371/journal.pone.0321739 (PMC12002523; doi:10.1371/journal.pone.0321739)
Supplement: S3 Appendix — (DOCX) [file pone.0321739.s003.docx]

**S3 Appendix**

**Comparisons of load force components on the moving object**

Here, we report the mean and variance of components of load force – inertial and spring forces – for each hand during object movement. Linear mixed-effects models were fitted with three experimental factors (*hand × task difficulty* *× load component)* and their two- and three-way interactions as fixed effects and participants are a random effect. Tukey-Kramer test was used to perform post-hoc pairwise comparisons when significant interaction effects were observed. Effect sizes were quantified by computing Cohen’s *d*. Significance was set at an α-level of 0.05.

The mean force (Fig S3A) showed a significant *hand* *× load component* interaction (F_(1,23)_ = 4.92, *p* = 0.03). Post-hoc analysis revealed that only the mean inertial force was higher in the right hand than the left hand (Cohen’s *d* =0.5). The mean force also showed a significant *task difficulty* *× load component* interaction (F_(1,23)_ = 223.0, *p* < 0.01). Post-hoc analysis revealed that the mean spring force was greater than the mean inertial force. However, the difference between the two mean forces was higher during irregular movement (Cohen’s *d* = 2.2).

The force variance (Fig S3B) showed a significant *hand* *× load component* interaction (F_(1,23)_ = 11.3, *p* < 0.01). Post-hoc analysis revealed that only the inertial force variance was higher in the right hand than the left hand (Cohen’s *d* =0.6). The force variance also showed a significant *task difficulty* *× load component* interaction (F_(1,23)_ = 77.7, *p* < 0.01). Post-hoc analysis revealed that the spring force variance was greater than the inertial force variance. However, the difference between the two force variances was higher during irregular movement (Cohen’s *d* = 3.3).


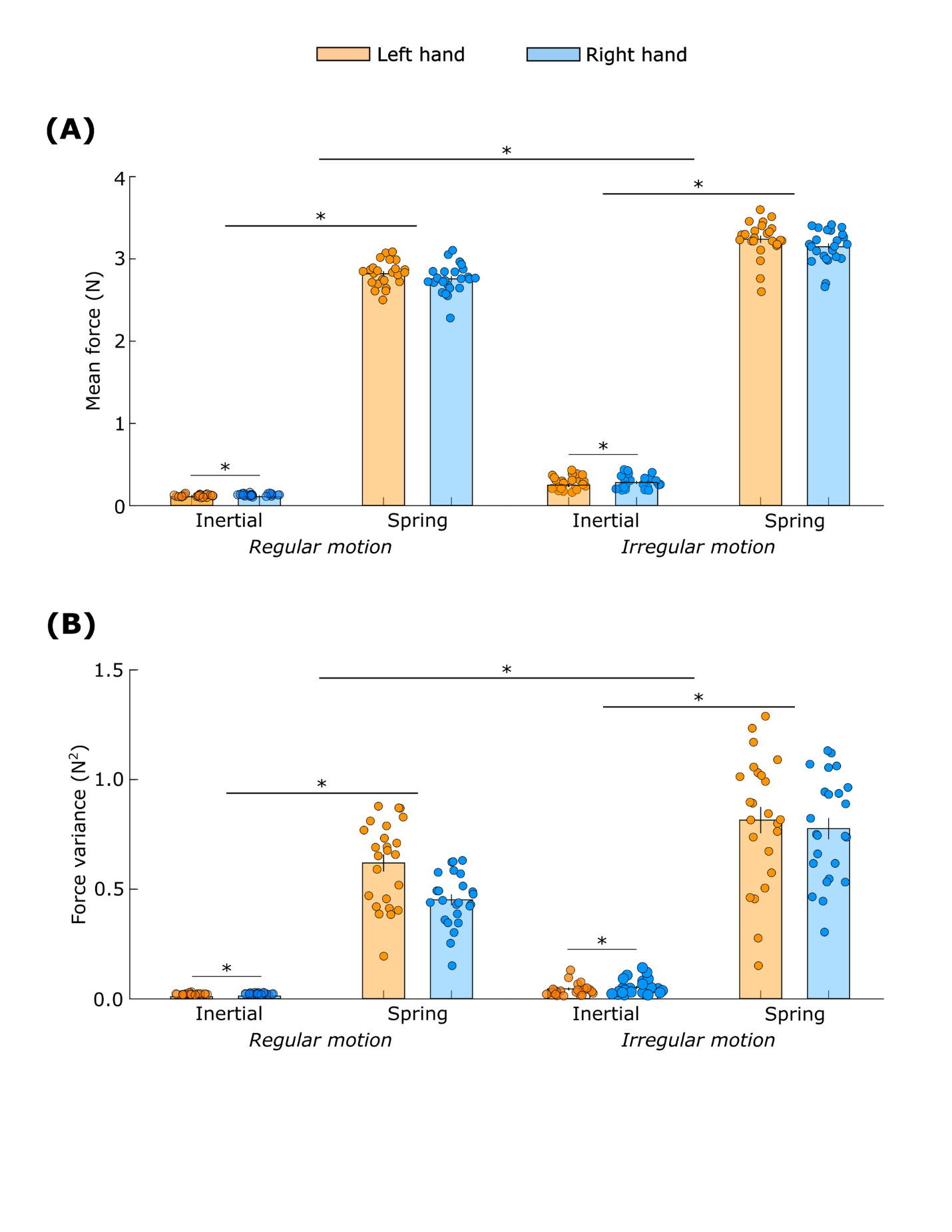


**Fig S3. Load force components**. (A) mean and (B) variance of forces. ‘*’ indicates significant differences (*p* < 0.05). Data are mean ± standard error.
