# Supplementary material for: Handy divisions: Hand-specific specialization of prehensile control in bimanual tasks
Source: PLoS One. 2025 Apr 16;20(4):e0321739. doi: 10.1371/journal.pone.0321739 (PMC12002523; doi:10.1371/journal.pone.0321739)
Supplement: S4 Appendix — (DOCX) [file pone.0321739.s004.docx]

**S4 Appendix**

**Performance scores**

Participants repeated the same movements 10 times in various tasks. Therefore, to check whether participants were able to predict the target’s position in each *task type*, a learning analysis was performed for each *hand* × *task type* block. To test whether learning occurred, a simple linear regression analysis was performed within each *hand* × *task type* block, with trial number as the independent variable and the score as the dependent variable. A significant negative slope would indicate learning occurred after repeated exposure to the target profile. The results are summarized in Table S4.

In the *left-hand* $\times$ *irregular task* block, the performance score improved in six participants. In the *right-hand* $\times$ *irregular task* block, the performance score improved in six participants. In the *left-hand* $\times$ *regular task* block, the performance score improved in four participants. In the *right-hand* $\times$ *regular task* block, the performance score improved in eight participants. Two participants demonstrated learning effects in three of the four tasks and four participants demonstrated learning effects in two of the four tasks.

Overall, only a minority of participants demonstrated learning in each of the tasks.

**Table S4. Summary statistics for the learning effect across trials in each *hand*** $\boldsymbol{\times}$ ***task type* block.** A *p-*value < 0.05 indicates slope that is significantly different than zero. A significant negative slope indicates an effect of learning and is denoted by ***.**

|  | *Left hand moving × irregular task* | | *Right hand moving × irregular task* | | *Left hand moving × regular task* | | *Right hand moving × regular task* | |
| --- | --- | --- | --- | --- | --- | --- | --- | --- |
| Participant # | Slope (cm^2^/trial) | *p-*value | Slope (cm^2^/trial) | *p-*value | Slope (cm^2^/trial) | *p-*value | Slope (cm^2^/trial) | *p-*value |
| 1 | 0.42 | 0.46 | 1.68 | 0.16 | 0.17 | 0.33 | 0.33 | <0.01 |
| 2 | -2.29* | 0.01 | -0.28 | 0.84 | 0.00 | 1.00 | -0.53* | 0.01 |
| 3 | -0.40 | 0.65 | -0.75 | 0.40 | 0.05 | 0.74 | -0.30 | 0.07 |
| 4 | -0.04 | 0.89 | 0.40 | 0.49 | -0.18 | 0.35 | 0.00 | 0.98 |
| 5 | 0.02 | 0.97 | -0.72 | 0.28 | -0.21 | 0.34 | -0.16 | 0.53 |
| 6 | -2.31 | 0.16 | -3.83 | 0.05 | -0.79* | 0.04 | -0.71 | 0.05 |
| 7 | -0.89 | 0.32 | -1.29 | 0.21 | 0.06 | 0.76 | -0.40 | 0.10 |
| 8 | -3.74* | 0.04 | -3.21* | <0.01 | -1.01* | <0.01 | -0.58 | 0.05 |
| 9 | -3.77* | 0.01 | -0.79 | 0.46 | -0.74* | <0.01 | -0.11 | 0.70 |
| 10 | -0.67 | 0.43 | -0.78 | 0.26 | -0.64 | 0.08 | 0.41 | 0.20 |
| 11 | -1.46 | 0.06 | -1.54 | 0.06 | -0.38 | 0.13 | -0.46 | 0.08 |
| 12 | -2.59 | 0.11 | -3.03 | 0.06 | -0.50* | <0.01 | -0.05 | 0.54 |
| 13 | -1.45* | 0.01 | -1.08* | <0.01 | -0.17 | 0.38 | -0.79* | <0.01 |
| 14 | 0.34 | 0.74 | -2.05* | 0.01 | -0.27 | 0.16 | -0.40 | 0.10 |
| 15 | -0.63 | 0.67 | -5.81* | <0.01 | -0.40 | 0.26 | -0.15 | 0.42 |
| 16 | -0.25 | 0.68 | -1.05 | 0.07 | -0.40 | 0.36 | -0.18 | 0.20 |
| 17 | -0.05 | 0.83 | -1.87* | <0.01 | -0.02 | 0.91 | -0.70* | <0.01 |
| 18 | -1.15* | 0.02 | -0.71 | 0.54 | 0.00 | 0.99 | -0.34 | 0.33 |
| 19 | -1.46 | 0.08 | -0.83 | 0.19 | -0.18 | 0.14 | -0.65* | <0.01 |
| 20 | -0.91 | 0.33 | 0.93 | 0.69 | -0.18 | 0.12 | -0.34 | 0.06 |
| 21 | -1.56 | 0.24 | -0.19 | 0.89 | 0.28 | 0.44 | -0.51* | 0.01 |
| 22 | -2.75* | 0.04 | -2.78* | 0.03* | -0.31 | 0.43 | -0.16 | 0.43 |
| 23 | -0.22 | 0.69 | -1.33 | 0.05 | -0.24 | 0.15 | -0.70* | 0.02 |
| 24 | -2.00 | 0.07 | -1.18 | 0.23 | -0.27 | 0.17 | 0.03 | 0.89 |
