# Supplementary material for: Handy divisions: Hand-specific specialization of prehensile control in bimanual tasks
Source: PLoS One. 2025 Apr 16;20(4):e0321739. doi: 10.1371/journal.pone.0321739 (PMC12002523; doi:10.1371/journal.pone.0321739)
Supplement: Attachment — Submitted filename: Responses to reviewer comments.docx [file pone.0321739.s006.docx]

We are grateful to the reviewers for their inputs. The manuscript is better because of their feedback.

All reviewer comments are addressed below. But we want to highlight three points about the revised version.

First: We have modified the term “dynamic dominance theory” to “complimentary dominance theory” throughout the manuscript based on a recent review paper (Kitchen et al 2024). Therefore, we use the term “complimentary dominance” here in the response document as well.

Reference:

Kitchen, N. M., B. Dexheimer, J. Yuk, C. Maenza, P. R. Ruelos, T. Kim and R. L. Sainburg (2024). "The complementary dominance hypothesis: a model for remediating the 'good' hand in stroke survivors." J Physiol.

Second: Although this was not suggested by the reviewers’, we created a ‘Limitations’ section that contains responses and improvements suggested by some of the reviewers’ comments.

Third: the line numbers mentioned in this document refer to those in the revised manuscript with changes tracked.

Journal requirements:

When submitting your revision, we need you to address these additional requirements.

1. Please ensure that your manuscript meets PLOS ONE's style requirements, including those for file naming. The PLOS ONE style templates can be found at

<https://journals.plos.org/plosone/s/file?id=wjVg/PLOSOne_formatting_sample_main_body.pdf> and

<https://journals.plos.org/plosone/s/file?id=ba62/PLOSOne_formatting_sample_title_authors_affiliations.pdf>

RESPONSE: Thank you for this comment. We have made changes based on these instructions.

2. Thank you for stating the following financial disclosure:

 [Anvesh Naik was funded by The Ross Fellowship awarded by Purdue University.].

Please state what role the funders took in the study.  If the funders had no role, please state: ""The funders had no role in study design, data collection and analysis, decision to publish, or preparation of the manuscript.""

If this statement is not correct you must amend it as needed.

Please include this amended Role of Funder statement in your cover letter; we will change the online submission form on your behalf.

RESPONSE: We have added this statement in the revised cover letter. Thank you for changing the online submission on our behalf.

3. We notice that your supplementary figures are uploaded with the file type 'Figure'. Please amend the file type to 'Supporting Information'. Please ensure that each Supporting Information file has a legend listed in the manuscript after the references list.

RESPONSE:

Thank you for spotting this. In the revision, we have not included the figure in the supporting material as a separate upload.

Furthermore, we have separated the single supplemental material file into its 4 components and uploaded those as separate files. This allows for better description of the contents.

We have included the Supporting Information legend after the reference list as instructed.

4. Please review your reference list to ensure that it is complete and correct. If you have cited papers that have been retracted, please include the rationale for doing so in the manuscript text, or remove these references and replace them with relevant current references. Any changes to the reference list should be mentioned in the rebuttal letter that accompanies your revised manuscript. If you need to cite a retracted article, indicate the article’s retracted status in the References list and also include a citation and full reference for the retraction notice.

RESPONSE: Thank you for this comment. We have checked the list and ensured that we have not cited retracted articles.

[Note: HTML markup is below. Please do not edit.]

Reviewers' comments:

Reviewer's Responses to Questions

**Comments to the Author**

1. Is the manuscript technically sound, and do the data support the conclusions?

The manuscript must describe a technically sound piece of scientific research with data that supports the conclusions. Experiments must have been conducted rigorously, with appropriate controls, replication, and sample sizes. The conclusions must be drawn appropriately based on the data presented.

Reviewer #1: Partly

Reviewer #2: Yes

2. Has the statistical analysis been performed appropriately and rigorously?

Reviewer #1: I Don't Know

Reviewer #2: Yes

3. Have the authors made all data underlying the findings in their manuscript fully available?

The [PLOS Data policy](http://track.editorialmanager.com/CL0/http:%2F%2Fwww.plosone.org%2Fstatic%2Fpolicies.action%23sharing/1/010f01930d75046b-26fa3a79-0c51-403c-b2b3-a25dfbc2696b-000000/z_7Pu7aHbqtZo5aYq4ckNMvCCKvigHxLmTAwsrb7Hs4=184) requires authors to make all data underlying the findings described in their manuscript fully available without restriction, with rare exception (please refer to the Data Availability Statement in the manuscript PDF file). The data should be provided as part of the manuscript or its supporting information, or deposited to a public repository. For example, in addition to summary statistics, the data points behind means, medians and variance measures should be available. If there are restrictions on publicly sharing data—e.g. participant privacy or use of data from a third party—those must be specified.

Reviewer #1: Yes

Reviewer #2: Yes

4. Is the manuscript presented in an intelligible fashion and written in standard English?

PLOS ONE does not copyedit accepted manuscripts, so the language in submitted articles must be clear, correct, and unambiguous. Any typographical or grammatical errors should be corrected at revision, so please note any specific errors here.

Reviewer #1: Yes

Reviewer #2: Yes

5. Review Comments to the Author

Please use the space provided to explain your answers to the questions above. You may also include additional comments for the author, including concerns about dual publication, research ethics, or publication ethics. (Please upload your review as an attachment if it exceeds 20,000 characters)

Reviewer #1: # General comments

\## Is the manuscript clearly written?
Overall, the manuscript is clearly written. It is easy to read for the experts, who are familiar with the terms and concepts used in the field. However, for the general scientific audience with no background in kinesiology (like myself) understanding the manuscript requires a fair amount of googling to learn the concepts that the authors use (e.g. "impedance", "load", "coupling", etc.). I find it perfectly normal that understanding a scientific paper requires a significant amount of expertise in the paper's domain; nevertheless, I think the authors can greatly increase the paper's appeal to a broader scientific audience by providing a brief introduction to the terms unfamiliar to the readers from outside the field. For example, a table with a few sentences briefly describing each term may be very useful.

RESPONSE: This is a very good suggestion. It forced us to think more carefully, particularly about impedance. We have included definitions of impedance, impedance control, and object-level impedance control in Table 1. The last term extends the long-used concept of ‘impedance control’ to object manipulation.

\## Is the experiment described in the manuscript technically sound?
The manuscript describes a rigorous and technically sound experiment for measuring several kinematic and kinetic variables (grip force, grip force-load force coupling, and various kinematic variables) and studying the influence of different experimental conditions on these variables. The statistical methods used by the authors generally seem reasonable to me, however I lack the expertise to make any definite comments on them.

\## Do the results presented in the manuscript support the conclusions?
This is my biggest beef with this manuscript. Whereas the authors do a good job of measuring the biomechanical variables of interest under several experimental conditions, their interpretation of the results of these measurements as evidence for the dynamic dominance theory is not convincing enough, at least for a reader like myself, who is not an expert in the field. In particular:

* The authors interpret the higher grip force in the stabilizing hand as the evidence for better impedance control. While this may be true, it's not immediately obvious. From a purely mechanical perspective, higher grip force itself does not impact the stability of instrumented object (as long as the force is sufficient to prevent the object from slipping from the hand). What matters for the object's stability is the efficient use of the corrective forces that the hand applies in response to the unexpected displacement of the object. I would expect a more efficient use of these corrective forces -- rather than the average grip force -- to be a sign of a better impedance control by a particular hemisphere.
It's worth noting that the authors suggest the wrist stiffness induced by the muscle co-contraction as a partial explanation for the link between the higher grip force and better impedance control. It is not obvious how this explanation supports the conclusion that that higher grip force indicates better hemispheric specialization for impedance control. If anything, one might argue that it actually __weakens__ the authors' conclusions by providing an alternative explanation.

RESPONSE:

Thank you for the comment. We agree with the reviewer that feedback-based corrections in grip force will impact the impedance of the object. But we suggest that, based on current evidence (cf. Hadjiosif and Smith, 2015; Naik and Ambike 2022), larger average grip force is also employed to increase the object’s impedance in dynamic and unpredictable situations.

We agree that exerting constant grip force on an object that is sufficiently high so that the digit-object friction is equal to the external force on the object will ensure a slip-free grasp. This is true in static conditions where the object is not being moved, and where the external load on the object is not changing. But when either of these conditions is not met, the net load on the object will change, and the grip force must compensate. The person can (1) modulate grip force so that it is always equal to (or greater than) the load, or (2) proactively increase grip force to a sufficiently high constant value so that it is greater than the minimum required to compensate for the largest expected load.

There is ample evidence that humans do both. When the load is predictable, we observe grip-load coupling. When the load is unpredictable, we observe higher mean grip force (Hadjiosif and Smith, 2015). In fact, humans always use a safety margin, even in static grasps, and this has been interpreted as an insurance policy against unexpected load fluctuations (cf. Westling and Johansson, 1984).

About muscle co-contraction: we do not agree that this is an alternative explanation. It is possible to increase co-contraction without increasing grip force; but we are saying that the increased grip force – *which we have observed* – must be accompanied by higher co-contraction (which we have not measured). A likely outcome of that is higher wrist joint stiffness which, in turn, contributes to higher object impedance (Franklin et al. 2003; Franklin et al. 2004). Therefore, we do not think that the co-contraction argument weakens our conclusions.

Put differently, our primary observation that supports superior impedance control by the left hand is the lower compliance of the object when it is stabilized by the left hand. Beyond that, we are offering mechanistic explanations, partially supported by our other data, of how this might be implemented by the body. We think that our explanations are consistent and our speculations are experimentally testable. We have added these descriptions from lines 475 to 492.

**References:**

Westling and Johansson, 1984. Factors influencing the force control during precision grip. Exp Brain Res, 53: 277-284

Hadjiosif AM, Smith MA. Flexible Control of Safety Margins for Action Based on Environmental Variability. J Neurosci. 2015;35(24):9106-9121.

Naik, A., & Ambike, S. (2022). Expectation of volitional arm movement has prolonged effects on the grip force exerted on a pinched object. *Experimental Brain Research*, *240*(10), 2607-2621.

Franklin DW, Osu R, Burdet E, Kawato M, Milner TE. Adaptation to stable and unstable dynamics achieved by combined impedance control and inverse dynamics model. J Neurophysiol. 2003;90(5):3270-3282. doi: 10.1152/jn.01112.2002. PubMed PMID: 14615432.

Franklin DW, So U, Kawato M, Milner TE. Impedance control balances stability with metabolically costly muscle activation. J Neurophysiol. 2004;92(5):3097-3105. Epub 20040616. doi: 10.1152/jn.00364.2004. PubMed PMID: 15201309.

* The authors assume that in their setup the participant will be able to track the moving target better when the tracking hand is controlled by a hemisphere specializing for predictive control; similarly, they assume that the subject will be able to stabilize the instrumented object better when the stabilizing hand is controlled by a hemisphere specializing in reactive control. It's worth noting that other publications (e.g. reference 12) rely on similar assumptions. Nevertheless, it's not clear to me why this assumption should hold. Two objects connected by a spring is a very simple mechanical system; to me it looks quite possible that the participants can learn to model it using the same predictive control mechanisms they use for modelling their hands. If this is the case, both tasks (tracking the target and stabilizing the object) require equal amount of predictive and reactive control; one cannot expect that participants will perform one task better when using a hemisphere specialized for predictive control and the other -- when using a hemisphere specialized in reactive control. The authors are clearly aware of this problem and discuss it in the "Discussion" section, e.g. lines 460 -- 465. Yet they fail to convincingly explain why their conclusions are not invalidated by such a possibility.

RESPONSE:

We are somewhat confused by this comment. We are not assuming that the hands will demonstrate different kinematic features. Rather, we explicitly evaluate whether this is true (see hypotheses H1 and H2).

Furthermore, we agree that two objects connected by a spring is a relatively simple system to model given the level of dexterity in humans. Also note that it is clear in the complementary dominance literature that both hemispheres use both predictive and impedance control mechanisms and so this will likely be the case for our tasks.

Nevertheless, there are key differences in the data – both kinematic and kinetic – in the way each hand performs the tasks, and these differences allow us to evaluate our hypotheses about complementary dominance.

According to complementary dominance theory, the two hemispheres are differentially specialized in predictive and impedance control mechanisms, and which mechanism is preferred by a hemisphere when controlling the contralateral hand is reflected in the motor performance of that hand. This is reflected in the data: we see superior tracking performance with the right hand and superior stability performance with the left hand.

\# Specific comments

* The code and data for the manuscript are available from doi:10.4231/GB7J-RA71. The zip archive downloaded through this DOI seems to contain both the data recorded by the authors and the code they used in their analysis. However, I failed to find within the archive any meaningful documentation explaining how the data is organized or how to use the code to reproduce their results. The lack of such documentation greatly reduces the usefulness of the code and data provided, and I would recommend the authors to provide a comprehensive documentation of their code and data if they have enough resources. Nevertheless, considering relative costs and benefits of properly documenting the code, I think this should be taken as a recommendation and not a mandatory requirement for publication.

RESPONSE: Thank you for this comment. We did have detailed documentation – a ReadMe file and a data dictionary that explain in detail the organization of the data and code. But, as the reviewer points out, it was not readily available.

Therefore, we have uploaded a new version to the repository (version 3). There are clear release notes on the landing page that tell the user where they will get the information to use the data and code. A ReadMe word document as well as a data dictionary are immediately available when the user downloads and unzips the data+code bundle from the repository. These files describe in detail the use of the code and the included data.

We have provided a new url to the latest upload with this revision.

* I recommend replacing the term "global dominance theory" with the "global hemispheric dominance theory" throughout the text to make the terminology more consistent with the literature.

RESPONSE: Thank you for the suggestion. We have implemented this change in the manuscript in line number 47. We have also modified the term “dynamic dominance theory” to “complimentary dominance theory” throughout the manuscript based on a recent review paper (Kitchen et al 2024).

References:

Kitchen, N. M., B. Dexheimer, J. Yuk, C. Maenza, P. R. Ruelos, T. Kim and R. L. Sainburg (2024). "The complementary dominance hypothesis: a model for remediating the 'good' hand in stroke survivors." J Physiol.

* I suggest replacing the heading "Equipment" (line 98) with "Instrumentation" or something similar.

RESPONSE: Thank you for the suggestion. We have used ‘Instrumentation’ to describe the section in line number 149.

* For the difficult condition it seems that the same sequence of brownian motion (mentioned in the lines 154 -- 155) was used within a single test subject (otherwise it would be impossible to compute meaningful std). Was the same sequence used for all the subjects? I recommend explaining it more clearly.

RESPONSE: Thank you for the suggestion. Yes, the same sequence was used in all Brownian motion trials for all subjects. We have added a description from lines 206 to 207.

* Caption for the Fig.5: I recommend explicitly emphasizing the fact that plots use different axis for the grip and load forces.

RESPONSE: Thank you for the suggestion. We have now mentioned this in the figure caption.

* Lines 281 and further down the text: I find the notation F(1,23) confusing. Can it be a copy-paste error? I suggest either correcting it or explaining it more clearly.

RESPONSE: Thank you for this comment. This is standard statistical notation widely used in motor control studies. This is an F-statistic for a given fixed effect (e.g., hand or task type). The numerator degree of freedom is 1. Because there are two levels in each fixed effect, the numerator degree of freedom is 2 – 1 = 1. The denominator degrees of freedom are 23. This is the number of observations minus the number of parameters estimated for the random effect, adjusted for the repeated measures design. Because there are 24 subjects, the denominator degrees of freedom are 24−1=23. This accounts for the variation between subjects in our mixed-effects model.

Since this is standard reporting practice, we did not alter or add any text in the manuscript.

* Line 293 and further down the text: I suggest adding the degree sign whenever reporting the angles.

RESPONSE: Thank you for this comment. We have made changes in the document. (Line 334 and caption of figure 7).

* I think that moving the technical details of CRQA to the appendix will make the manuscript easier to read.

RESPONSE: This is a valuable suggestion. We have shifted the technical description for CRQA into supporting information Appendix S1 and shortened the cross-recurrence quantification section in methods from lines 287 to 298.

Reviewer #2: Thank you for the opportunity to review the manuscript by Naik and Ambike entitled “Handy divisions: Hand-specific specialization of prehensile control in bimanual tasks”.

The aim of this study was to investigate whether object manipulation supports the dynamic dominance theory. This theory suggests that the left hemisphere specializes in predictive control, making the right hand more adept at coordinated movement, while the right hemisphere specializes in impedance control, making the left hand better at stabilization. Previous studies using wrist kinematics have supported this theory and this work seeks to utilize object orientation and grip force measures.
The authors designed an instrumented object for each hand, linked by a spring, that measured pinch force and position. The position of each object was represented by cursors on a screen. Participants were instructed to maintain one hand’s position within a stationary target while using the other hand to track a moving target. The moving target followed either a predictable or irregular pattern, and participants completed trials with either hand serving as the stabilizer or the tracker.
Performance measures included basic performance metrics, task-specific measures, and grip force control measures. The findings showed that the right hand excelled in tracking performance, while the left hand was better at stabilization. They also observed stronger grip-load coupling—a marker of predictive control—in the right-hand during object movement, while mean grip force—a marker of impedance control—was stronger in the left-hand during stabilization. Task difficulty did not significantly impact force characteristics.

The authors are to be commended on the overall rationale, design and presentation of their study. The manuscript was well written, and the figures were well presented.
Main Comments
1. My primary concern with this study is the absence of left-hand dominant participants and the lack of neuroimaging data, both of which impact the strength of conclusions about neurophysiology and hemispheric specialization. While the authors do connect their findings to previous studies, without data from left-hand dominant participants or neuroimaging, it’s challenging to make definitive connections between observed behaviors and neurophysiological mechanisms.

a. Including the discussion of limitations around the exclusion of left-hand dominant individuals is valuable. However, the authors also reference prior work showing distinct neural organization for left-hand dominant individuals, which seems to contradict their generalized claims about hemispheric specialization earlier in the paper. This discrepancy highlights the need for a more cautious interpretation of hemispheric specialization, as it appears to depend on hand dominance.

RESPONSE: Thank you for this comment. This is a valid point. Our work focuses on right-handed persons and we have modified the language in the manuscript to highlight that our findings are limited to this population only from lines 431 to 432. We have also emphasized this as a limitation in the ‘Limitations’ section on line 551 and lines 560 to 561. We also mention on line 560 and 561 that study of left handers and ambidextrous individuals is essential to develop a more comprehensive theory.

Furthermore, we have also clarified that our work is strictly behavioral and neurophysiological data is needed to claim that the theory extends to prehension (lines 447 to 448 and lines 581 to 584). We also mention the need for such data at the end of the abstract and the conclusion (lines 40 to 42 and lines 600 to 601).

b. (Lines 394-399) Brain structures are listed to emphasize the fact that arm motion and digit forces/grasping control is controlled by distinct structures. However, dorsal premotor cortex is included for both. I would suggest emphasizing the distinct neural pathways in the anterior and medial intraparietal areas.
i. Lines 403-404 then go on to say that reaching and grasping neural circuits interact. I would suggest clarifying language because the dissociation discussed in one paragraph seems to contradict the interaction in the next paragraph.

RESPONSE: Thank you for the valuable suggestion. We have clarified this point in lines 431 to 448.

2. Figure 7 indicates that, regardless of task difficulty or whether the task involves tracking or stabilizing, the right hand consistently shows reduced mean tilt. Since mean tilt is used here as a measure of stabilization, this finding seems counter to the hypothesis that the right hemisphere, and thus the left hand, would specialize in stabilization. I suggest further elaboration on this unexpected result and its implications, as it does not appear to support the main hypothesis.

RESPONSE: Thank you for this suggestion. However, we did not provide feedback based on the object’s tilt. For this reason, we did not consider mean tilt as a measure of stabilization. Rather, the data were provided to demonstrate that the tilt was small, and therefore it does not significantly impact the computation of grip force. Since the object’s tilt did not affect the task goal, it is difficult to say that the result contradicts the hypothesis. We have added this in Methods from lines 258 to 261, and we have added the interpretation of the tilt in the Limitations section on lines 575 to 580.

Minor Comments
1. Vague language used in introduction: “dominant (right) hand is better…” (line 4), “superiority of the left hemisphere…” (line 6)

RESPONSE: Thank you for this suggestion. We have made changes to lines 47, 48, and 50. The new language is slightly less vague, but this is our stylistic preference. We prefer to introduce the reader to the overarching ideas, and then proceed to provide details in later paragraphs.

2. Elaborate on inclusion/exclusion criteria to indicate whether individuals with any motor or sensory impairments were excluded

RESPONSE: Thank you for pointing this out. We have added this information on lines 142 and 143.

3. Add the icons used in figure 5 to indicate which object moved and which remained stationary to figure 6

RESPONSE: Thank you for this comment. However, the Fig 6 is cross recurrence plots for grip-load coupling. Since this analysis was done only on the moving hand, we did not include the moving and static object icon used in Fig 5. We have added this note in the caption for Fig 6.

4. Figure 9D has smaller points than the other subplots

RESPONSE: Thank you for pointing this out. We have edited the figure.

Overall, I think this is a well written manuscript and highlights important results. The minor comments should be resolved, and the neurophysiological conclusions drawn from behavioral results and prior literature without imaging data related to the study participants should be minimized.

RESPONSE: This is an important point. We now indicate that the behavioral evidence points must be followed up with imaging work – just like previous work with reaching movements in persons with stroke. We have created a ‘Limitations’ section. There we retain the text on left handed individuals, and we also add the lack of neuro-imaging data as a limitation.

6. PLOS authors have the option to publish the peer review history of their article ([what does this mean?](https://track.editorialmanager.com/CL0/https:%2F%2Fjournals.plos.org%2Fplosone%2Fs%2Feditorial-and-peer-review-process%23loc-peer-review-history/1/010f01930d75046b-26fa3a79-0c51-403c-b2b3-a25dfbc2696b-000000/hs2E8XSeBT-3-ZUF8oBT-GiySdUZ6UtV_rRvlssVlUU=184)). If published, this will include your full peer review and any attached files.


If you choose “no”, your identity will remain anonymous but your review may still be made public.


**Do you want your identity to be public for this peer review?** For information about this choice, including consent withdrawal, please see our [Privacy Policy](https://track.editorialmanager.com/CL0/https:%2F%2Fwww.plos.org%2Fprivacy-policy/1/010f01930d75046b-26fa3a79-0c51-403c-b2b3-a25dfbc2696b-000000/EopE_HKBhZvJFHEDsq_loFWwbbogApasj4OkyJaPsUI=184).

Reviewer #1: No

Reviewer #2: No
